# Primary hepatic neuroendocrine tumours—Case series of a rare malignancy

**DOI:** 10.1016/j.ijscr.2019.01.025

**Published:** 2019-01-30

**Authors:** Nelson Chen, Kellee Slater

**Affiliations:** a Department of Hepatobiliary and Pancreatic Surgery, Princess Alexandra Hospital, 199 Ipswich Road, Woolloongabba, Queensland, 4102, Australia; b University of Queensland, Australia; c Department of Hepatobiliary and Pancreatic Surgery, Greenslopes Private Hospital, Newdegate Street, Greenslopes, Queensland, 4102, Australia

**Keywords:** Hepatobiliary cancers, Primary liver cancers, Primary hepatic cancers, Neuroendocrine tumours, Primary hepatic neuroendocrine tumours, Case series

## Abstract

**Introduction:**

Primary hepatic neuroendocrine tumours (PHNET) were first described by Edmondson et al. in 1958 and are rare, accounting for only 0.3% of all neuroendocrine tumours. Only several hundred cases have been reported.

**Presentation of case:**

We present two cases. The first is a 65-year-old asymptomatic male referred with a liver lesion on ultrasound performed to investigate a mildly elevated Alanine Aminotransferase (ALT). Hepatitis serology and tumour markers were normal. He had an unremarkable colonoscopy and gastroscopy. CT and MRI revealed a single liver lesion adjacent to the gallbladder suspicious for malignancy. He underwent a segment IVb/V liver resection. Histology was consistent with a 65 mm grade 2 PHNET. Subsequent Dotatate PET/CT scans have been normal at 5 years. The second is an asymptomatic 73-year-old male referred with fluctuating hepatic enzymes and a history of alcohol overuse. Imaging revealed a suspicious lesion in segment III of the liver. He underwent a left lateral liver resection. Histology revealed an 18 mm grade 1 PHNET. A subsequent Dotatate PET/CT was normal with no new disease at six months.

**Discussion:**

PHNET, albeit rare are in the differential diagnosis for primary hepatic malignancies. Tumour markers are usually normal and radiological imaging can mimic other hypervascular hepatic tumours. Surgery is the only curative treatment for localised disease to date.

**Conclusion:**

PHNET needs to be considered in asymptomatic patients with hypervascular hepatic lesions. More research is required before other adjunct treatment options can be suggested.

## Highlights

• Primary hepatic neuroendocrine tumours are rare.
• Liver enzymes and tumour markers are often normal.
• Radiological imaging may mimic other hypervascular liver tumours.
• Surgical resection is the only curative treatment for localised disease.

## 1 Introduction

Neuroendocrine Tumours (NETs) are rare and mainly arise in the gastrointestinal tract (GIT), pancreas and tracheobronchopulmonary system [1]. It represents only 1–2% of all GIT malignancies and mostly occur in the small bowel [2]. The liver is the most common site for metastatic NETs [3], however, primary hepatic NETs (PHNET) are rare, accounting for only 0.3% of all NET cases [2]. PHNETs were first described by Edmondson et al. in 1958 [3] and only several hundred cases have been reported in literature [4]. Diagnosis is difficult because investigations are non-specific and often negative, and imaging mimic other vascular hepatic tumours. We hereby present our experience with two cases of PHNET, reported in line with the PROCESS criteria [5].

## 2 Methods

Registration and Ethics: the case series is registered on Research Registry (UIN: researchregistry4623). Ethics approval from the hospital’s Human Research and Ethics Committee (HREC) was obtained (protocol 18/43). All procedures performed were in accordance with the ethical standards of the institutional and the 1964 Declaration of Helsinki and its later amendments. Written informed consent was obtained from the patients for publication of this case series and accompanying images. A copy of the consent is available for review by the Editor-in-Chief of this journal on request.

### 2.1 Study design

The case series is a single-centered, retrospective and consecutive in design.

### 2.2 Setting

The patients were managed in a private practice setting.

## 3 Case series

### 3.1 Case 1

A 65-year-old man was referred in April 2013 with an echogenic segment IVa lesion found incidentally on ultrasound scan (USS). He was being investigated for fatty liver disease due to an elevated ALT (45 U/L). The patient consumed one bottle of wine daily with a background of hypertension, dyslipidaemia and gastroesophageal reflux disease. The patient was asymptomatic with a normal abdominal examination. His bloods and tumour markers including carcinoembryonic antigen (CEA), cancer antigen (CA) 19-9 and alpha-fetoprotein (AFP) were normal. A colonoscopy and gastroscopy was performed revealing only non-specific duodenitis with no malignancy.

A triple phase computed tomography (CT) scan identified a bilobed exophytic 70 × 56 x 78 mm segment IV liver lesion adjacent to the gallbladder and displacing the cystic artery. There was heterogenous hyperenhancement during arterial phase with washout in portal venous (PV) phase and no delayed enhancement. A magnetic resonance cholangiopancreatography (MRCP) showed the lesion had a multi-cystic or necrotic component posteriorly measuring 18 mm and was hyperintense in both T1 and T2-weighted imaging (WI) without fat or calcification. There was arterial enhancement that persisted into the PV phase without delayed phase enhancement. The right portal vein and its segmental branches were draped around the lesion causing mild compression, while the segmental biliary tree distal to the mass was dilated. The patient did not undergo a pre-operative biopsy. The differential diagnosis was hepatocellular carcinoma (HCC) in a non-cirrhotic liver, lymphoma or primary gallbladder tumour.

**Fig. 1:**
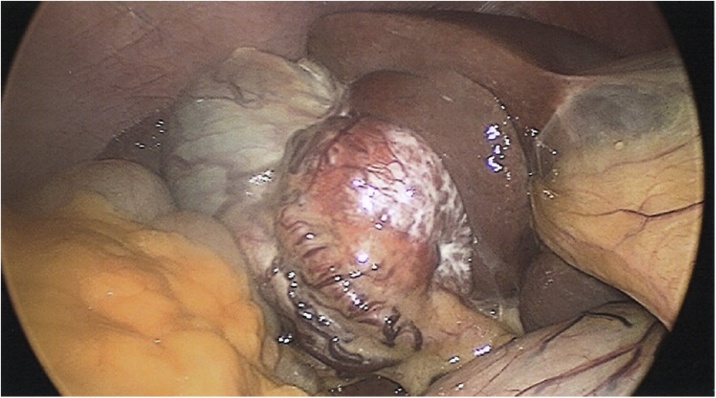
Mass displacing the gallbladder in patient 1.

**Fig. 2:**
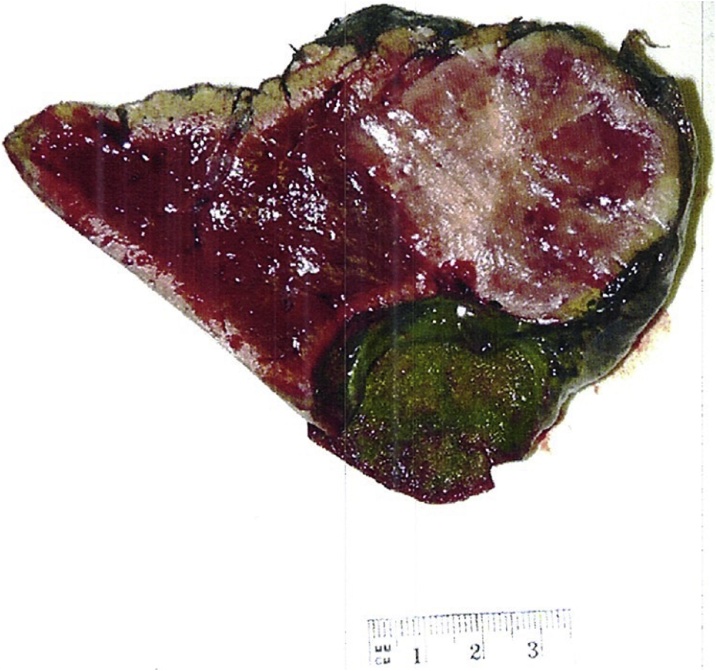
Well-delineated 64 × 40 × 59 mm mass adjacent to gallbladder in patient 1.

The patient underwent a segment IVb/V liver resection with enbloc cholecystectomy by a specialist hepato-pancreato-biliary (HPB) surgeon. The small bowel was normal. The mass is illustrated in Fig. 1. Macroscopically, there was a well-defined solid mass measuring 64 × 40 × 59 mm (Fig. 2). Microscopically, the tumour demonstrated an insular, acinar and trabecular architecture with variably fibrotic stroma. There was nuclear pleomorphism, readily identifiably mitoses and a Ki67 proliferation index of 3.4%. Immunohistochemical (IHC) stains were positive for synaptophysin, CD56 and weakly for CDX2 consistent for a grade 2 PHNET. IHC stains for chromogranin, gastrin and TTF-1 were negative.

The patient underwent a Dotatate positron emission tomography (PET)-CT scan post-operatively which revealed mild uptake in the head and uncinate process of the pancreas. This was considered to be a normal physiological variant given a normal CT scan pre-operatively. The Dotatate PET-CT scan was repeated six months later revealing a similar but stable appearance in the pancreas. Annual follow-up with CT scans for the last five years have been reassuring with no signs of recurrence or metastatic disease.

### 3.2 Case 2

A 73-year-old male was referred with mildly deranged liver enzymes. The patient was a heavy drinker, consuming 100 g of alcohol per day for four decades. He had a background of heterozygous haemochromatosis, pacemaker and a transurethral resection of the prostate. The patient was asymptomatic with a normal examination. His hepatocellular liver enzymes were minimally elevated initially but had normalised on repeat testing a month later. Tumour markers including AFP, CA 19-9 and CEA were all normal. The patient had a normal colonoscopy two years prior.

An USS revealed hepatomegaly with a solid 25 mm hypoechoic area in segment III with a cystic component. The CT scan showed a 20 mm segment III liver lesion with enhancement in arterial and PV phase and washout on the delayed phase with an enhancing capsule. Due to the alcohol history the possibility of HCC in a cirrhotic liver was suspected. After a satisfactory indocyanine green clearance test, the patient underwent an uncomplicated left lateral liver resection by the same specialist HPB consultant surgeon. The patient was not macroscopically cirrhotic and there were no lesions in the small bowel. The tumour measured 18 × 17 mm. Microscopically, there was mild hepatic steatosis with no fibrosis. IHC staining for synaptophysin and chromogranin were positive. There were no mitoses but a Ki67% proliferation index of 0.5% was consistent with a well-differentiated grade 1 NET. IHC stains for HepPar 1, CD10, TTF-1, CDX2 and PAX8 were negative.

The patient underwent a Dotatate PET-CT scan two months post operatively to search for an undiagnosed primary and this was normal. Repeat Dotatate PET-CT 6 months later was again normal. Given the patient had a colonoscopy only two years prior and there were no other CT or PET-CT signs of extra-hepatic disease, a final diagnosis of HPNET was made.

## 4 Discussion

PHNETs are more frequent in females (58.5%) [6] with a mean age of occurrence of 49.8 years [6]. Patients are often asymptomatic and referred with incidental findings. Symptoms may include right-sided abdominal pain, distension, fatigue, weight loss or obstructive jaundice [7] and only 6.8% of patients [3] present with signs of carcinoid syndrome such as flushing, diarrhoea, abdominal pain and palpitations [3,7]. Bloods and tumour markers such as AFP, CEA, CA 19-9 and CA125 are often normal or non-specific and have no diagnostic value even when elevated [3,8]. Serum chromogranin A (CgA) has a sensitivity of 87–100% and specificity of 92% in the diagnosis of NET and may be a useful non-invasive marker to monitor recurrence [8], but is often overlooked in the workup because PHNETs are rare.

The diagnosis of PHNET requires a histopathological specimen as well as exclusion of an extrahepatic primary lesion [9] because the liver is the most common site for metastasis of GIT NETs [10] and a rare site for a primary NET to occur. Pre-operative diagnosis of PHNET is difficult because radiological findings can mimic other hypervascular hepatic lesions such as HCC, cholangiocarcinomas [3], focal nodular hyperplasia [11] and haemangiomas [8].

USS of PHNETs can appear hypoechoic, hyperechoic or mixed in appearance. Li et al. reported that 60% of their case series displayed intralesional blood vessels compared to only 26–28% in metastatic liver NETs [12]. CT scans frequently show arterial enhancement with PV washout [12] which is a typical pattern often seen in HCC [11]. Magnetic resonance imaging (MRI) scans tend to demonstrate lower apparent diffusion coefficient (ADC) values compared to the normal surrounding liver. Lesions are predominantly hypointense in T1WI and hyperintense in T2WI [11]. PET-CT scans are mostly performed post-operatively after histological diagnosis of NET to exclude extra-hepatic sites. They are also more sensitive than somatostatin scans in the diagnosis of NET [4] which can only provide limited information about the aggressiveness of the tumour and can miss NETs that have limited somatostatin receptor sites [13].

Macroscopically, PHNETs appear as solid lesions that can have cystic components [7] with a gray-yellow [7] or brown [4] colour. Tumour margins can be well or poorly-circumscribed [7] and may display haemorrhagic areas. Haematoxylin and Eosin stains classically show trabecular, glandular or solid cell patterns but is not specific for PHNET [7]. IHC staining typically show positivity to CgA (66.7–95% immunoreactivity) [3,13], neuron-specific enolase (74.1–90% immunoreactivity) [13,14] and synaptophysin (48.9–91.7% immunoreactivity) [13] as well as CD56 and cytokeratin [15].

Surgery is currently the only curative treatment for localised PHNET with 5-year survival rates ranging from 74 to 78% and a recurrence rate of 18% [16]. Liver transplant can be considered if there are multiple hepatic lesions or if the lesion is too large without adequate liver reserve [6]. The use of transcatheter arterial chemoembolisation (TACE) for cytoreduction prior to surgery and the use of chemotherapy for poor operative candidates have been reported, but their effectiveness is questionable [13]. Somatostatin analogues such as octreotide have been considered by reducing hormone release and stunting tumour growth. However, PHNETs are classically endocrinologically silent so their use has not been well demonstrated [9]. The strength and limitation of this study is the number of participants and despite being a rare malignancy, we were able to identify two cases. However, due to the small study size, little conclusions are able to be made further about this malignancy.

## 5 Conclusion

Little is known about the clinical course of PHNETs because of the scarcity of cases. Bloods and tumour markers are non-specific and radiological findings mimic other hypervascular hepatic lesions. More studies are needed to investigate the effectiveness of TACE and chemotherapy on PHNETs but this may be difficult given the rarity. PHNETs should be considered in the diagnosis and management of all hypervascular hepatic masses, especially in patients with no chronic liver disease and normal tumour markers.

## Conflicts of interest

Nil.

## Sources of funding

Nil.

## Ethics approval

Ethics approval from the hospital’s Human Research and Ethics Committee (HREC) was obtained (protocol 18/43). All procedures performed were in accordance with the ethical standards of the institutional and the 1964 Declaration of Helsinki and its later amendments.

## Consent

Written informed consent was obtained from the patients for publication of this case series and accompanying images. A copy of the consent is available for review by the Editor-in-Chief of this journal on request.

## Author’s contribution

Nelson Chen: original draft, review and editing, final approval of version to be submitted.

Kellee Slater: conceptualisation, review and editing, revising critically for important intellectual content and final approval of version to be submitted.

## Registration of research studies

Name of research: Research Registry.

Date of registery: 11 January 2019.

URN: researchregistry4623.

## Guarantor

Nelson Chen and Kellee Slater.

## Provenance and peer review

Not commissioned, externally peer-reviewed.
